# HIF- and Non-HIF-Regulated Hypoxic Responses Require the Estrogen-Related Receptor in *Drosophila melanogaster*

**DOI:** 10.1371/journal.pgen.1003230

**Published:** 2013-01-31

**Authors:** Yan Li, Divya Padmanabha, Luciana B. Gentile, Catherine I. Dumur, Robert B. Beckstead, Keith D. Baker

**Affiliations:** 1 Department of Biochemistry and Molecular Biology and the Massey Cancer Center, Virginia Commonwealth University School of Medicine, Richmond, Virginia, United States of America; 2 Department of Pathology, Virginia Commonwealth University School of Medicine, Richmond, Virginia, United States of America; 3 Department of Poultry Science, University of Georgia, Athens, Georgia, United States of America; University of California San Francisco, United States of America

## Abstract

Low-oxygen tolerance is supported by an adaptive response that includes a coordinate shift in metabolism and the activation of a transcriptional program that is driven by the hypoxia-inducible factor (HIF) pathway. The precise contribution of HIF-1a in the adaptive response, however, has not been determined. Here, we investigate how HIF influences hypoxic adaptation throughout *Drosophila melanogaster* development. We find that hypoxic-induced transcriptional changes are comprised of HIF-dependent and HIF-independent pathways that are distinct and separable. We show that normoxic set-points of carbohydrate metabolites are significantly altered in *sima* mutants and that these animals are unable to mobilize glycogen in hypoxia. Furthermore, we find that the estrogen-related receptor (dERR), which is a global regulator of aerobic glycolysis in larvae, is required for a competent hypoxic response. dERR binds to dHIFa and participates in the HIF-dependent transcriptional program in hypoxia. In addition, dERR acts in the absence of dHIFa in hypoxia and a significant portion of HIF-independent transcriptional responses can be attributed to dERR actions, including upregulation of glycolytic transcripts. These results indicate that competent hypoxic responses arise from complex interactions between HIF-dependent and -independent mechanisms, and that dERR plays a central role in both of these programs.

## Author Summary

When oxygen levels fall below normal, cells are said to be in a hypoxic state. Once in hypoxia, dramatic changes are induced that allow for adaptation. In particular, energetic metabolism and transcription are highly affected. HIF (hypoxia inducible factor) is a highly conserved factor that is the driving force behind many hypoxia-induced changes—it is inactive in normal conditions and becomes active in hypoxia. Using the fruit fly as a model system, we show that hypoxic responses consist of HIF and non-HIF-dependent pathways. These response programs counteract the impacts of low oxygen by broadly influencing different cellular processes such as the breakdown of sugars, but only at appropriate developmental times. We provide evidence that HIF- and non-HIF-dependent pathways are complemented by the actions of the steroid hormone receptor estrogen-related receptor (ERR), which we show is also essential in hypoxia. Our results place new emphasis on the actions of HIF and suggest that alternative HIF-independent pathways play a more prominent role than previously thought.

## Introduction

The ability to adapt to limiting oxygen requires metabolic versatility, with cells transitioning toward glycolytic lactate production for energy production. Complementing this strategic change of metabolism are complex shifts in the transcriptome, which add durability to the initial hypoxic response. At the vanguard of the transcriptional reply to hypoxia is the HIF transcriptional complex, which is comprised of the oxygen-labile hypoxia-inducible factor-1a (HIF-1a) and its stable partner HIF-1b. This ancient pathway is central to the hypoxic response and is highly conserved from worms to human [1]. The actions of the HIF complex exert considerable influence in the etiologies of many diseases, including cancers and heart disease [2]–[4]; these conditions have a hypoxic component – and therefore an altered metabolic component – that is critical to disease progression.

In normoxia (N), HIF-1a is marked by a set of 2-oxoglutarate-utililizing prolyl hydroxylases (PHDs) that recognize specific proline residues within the oxygen-dependent degradation (ODD) domain [5], [6]. Prolyl modification of HIF-1a allows it to associate with the von Hippel-Lindau (VHL) tumor-suppressor and it is subsequently degraded [7]–[10]. Hypoxia disrupts the degradative cascade, allowing HIF-1a accumulation and activation of the HIF transcription pathway [11], [12].

The number of transcripts impacted by HIF-1a is large and ontologically diverse. Despite this, a few affected pathways generally characterize HIF-mediated adaptation responses, including upregulation of angiogenic [13], [14], erythropoietic [15], and glycolytic transcripts [16], [17]. The total hypoxic response, however, is not entirely dependent on the HIF pathway. For example, Shen et al. found 110 hypoxia response genes in *C. elegans*, 47 of which were induced in the absence of HIF [18]. Although HIF-independent hypoxia-induced activities have also been identified in other organisms, these pathways remain poorly understood, though even in mammalian cells, HIF-1a is dispensable for hypoxic upregulation of a host of transcripts [19]–[21]. These results suggest that HIF-independent hypoxic signaling mechanisms may act in concert with, or even supplant, the HIF response pathway in a context-dependent manner.

*Drosophila melanogaster* deal with no/low oxygen conditions well when compared to mammals, and can survive anoxic challenge for hours at a time [22], [23]. Flies maintain the three fundamental components of the HIF pathway: 1) the HIF prolyl hydroxylase (Fatiga); 2) dVHL; and 3) both components the HIF complex – dHIFa (encoded by *sima*) and Tango (dHIFb) [24]. As in mammals, dHIFa has an ODD domain that is sufficient to direct oxygen-sensitive degradation when hydroxylated [25]. While previous studies have examined hypoxic responses in adult flies [26], [27], the precise input that dHIFa has in this process has not been examined. In contrast, detailed studies have shown that dHIFa plays a vital role in directing hypoxia-driven terminal branching of the tracheal system during development [28], [29]. The *Drosophila* tracheal network serves as the fly respiratory system, and it is noteworthy that its developmental branching bears a striking resemblance to processes controlling mammalian angiogenesis [30]. In addition, similar hypoxia-induced metabolic transitions have been reported in flies and mammals [31], although these remain poorly defined.

The highly conserved dERR nuclear receptor directs a developmentally-regulated transcriptional switch towards glycolytic metabolism that supports developmental growth [32]. This function is akin to that described for ERRa in vertebrates, which is associated with glycolytic metabolism and breast cancer [33]–[38]. Importantly, mammalian ERRs are also active participants in HIF-mediated hypoxic responses. They are directly recruited by HIF-1a to HREs and are required for a complete transcriptional response at specific promoters [39], suggesting that ERRs play a critical role in hypoxic responses.

We set out to interrogate hypoxic responses in *Drosophila* and wanted to assess the influence of dHIFa on transcriptional and metabolic adaptation. We report here that the hypoxic transcriptional response segregates into distinct HIF-dependent and HIF-independent pathways. These pathways are differentially sensitive to hypoxic challenge in a temporal fashion during development, but both pathways are most sensitive prior to metamorphic onset and least active in the immediate hours following pupariation. Contrary to expectations, we find that upregulation of glycolytic transcripts is non-HIF-dependent. Our metabolic analysis suggests that loss of dHIFa has a profound and wide-ranging affect on all aspects of carbohydrate catabolism when unchallenged in normoxia. In hypoxia, however, *sima* mutants remain unable to mobilize glycogen, which is preferentially depleted under hypoxic conditions. We additionally show that dERR is required during hypoxia, in that it controls a unique set of hypoxia-regulated dERR-dependent transcripts that include HIF-independent glycolytic genes. Altogether, our studies raise important questions regarding the breadth of HIF involvement in hypoxic transitions and identify dERR as an essential factor that complements HIF-dependent and -independent responses.

## Results

### Robust transcriptional response to hypoxia in late third instar larvae

**Figure 1:**
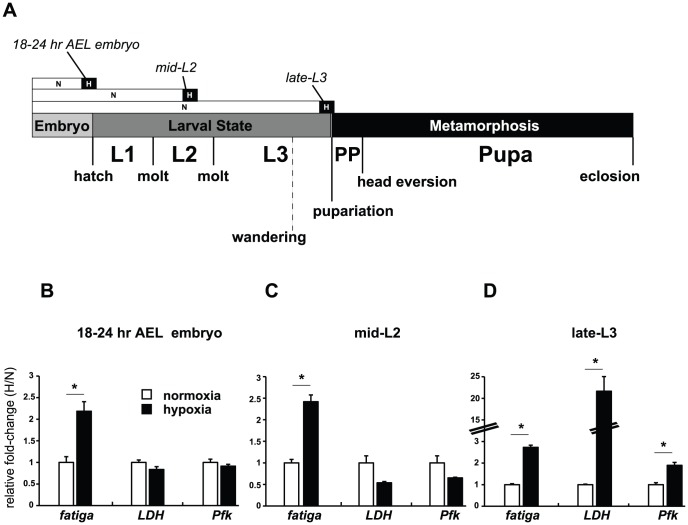
Temporal-dependent hypoxic responses. (A) Hypoxic treatment regimen of *w^1118^* animals that were allowed to develop in normoxia (N) until they reached one of three developmental stages, at which point they were treated for 6 hours in N or hypoxia (H) (4% O_2_). (B–D) qRT-PCR analysis was performed to assess the expression of *fatiga*, *LDH*, and *Pfk* at 18–24 hr AEL, mid-L2, or partial clear-gut larvae in late-L3. All experiments were performed in triplicate from pools of biological replicates. Values are normalized to *rp49* expression and are reported as the relative fold-change of H/N. Error bars are the SEM. * = *p*-value<0.05.

To better understand the contribution of in the hypoxic adaptation response, we wanted to determine the developmental time point when dHIFa was most active. To start, we examined the wild-type expression of two known hypoxia-responsive transcripts in *Drosophila*, *lactate dehydrogenase* (*LDH*, known also as: *ImpL3*, *CG10160*), and the HIF prolyl hydroxylase, *fatiga* (*CG31543*) [40], [41]. We also examined the rate-limiting enzyme of glycolysis, phosphofructokinase, encoded by *Pfk* (*CG4001*) as a potential hypoxia-responsive gene. We surveyed three times points, late embryo, mid-second instar (mid-L2) larvae, and late-L3 larvae by qRT-PCR to examine transcriptional responses of whole animals that were allowed to develop in normoxia and then challenged with a 4% O_2_ treatment for 6 hours – hereafter referred to as H-treatment (Figure 1A). This level of oxygen, and this time course, has previously been shown to mobilize the fly HIF pathway [42]. As seen in Figure 1D, the late-L3 time point of wandering larvae [−10 to −4 hours relative to the onset of pupariation (RTP)] is a period where each of the three genes is significantly induced by H-treatment. This expression profile is different from responses observed in embryos 18–24 hr after egg laying (AEL) (Figure 1B) and mid-L2 larvae (Figure 1C), when *LDH* and *Pfk* were unresponsive to treatment, indicating that hypoxic responses are developmentally tempered.

### Identification of HIF-dependent and HIF-independent hypoxic transcripts

To establish the identity of the full complement of H-regulated transcripts, RNA samples were prepared from N- and H-treated pools of control *w^1118^* animals and *sima* mutants at the late-L3 time. The *sima* mutant line (*sima^07607^*) contains a lethal P-element insertion in the first intron in the *sima* locus, which eliminates detectable expression of the transcript, rendering the animals incapable of directing expression of an oxygen-sensitive murine *LDH*-reporter and, importantly, unable to respond competently to hypoxic challenge [42], [43].

As expected, H-treatment resulted in a pronounced change in the transcriptome. Using the microarray scheme outlined in Figure S1, we extracted a series of significantly altered gene sets (Table S1). We were primarily concerned with identifying two mutually exclusive H-regulated gene sets – HIF-independent (HI) and HIF-dependent (HD). Transcripts that did not exhibit at least a 1.5-fold change in expression, and which did not have a false discovery rate (FDR, *q*-value) of less than 1%, were not included in any set. This high stringency means that we have likely excluded genuinely H-regulated transcripts from our final sets, be they HD or HI. Despite this, we classified 254 transcripts as HI and 171 as HD. It is important to note that the HI and HD categorizations reflect the hypoxic responsiveness of individual transcripts at the late-L3 time alone. The top 20 affected transcripts from the HIF-dependent and -independent categories are listed in Table 1.

**Table 1:** List of 20 top transcripts whose expression changes in response to hypoxic challenge in a dHIF-dependent or -independent fashion.

Top 20 HIF-Dependent Hypoxia Response Genes					
Probe Set ID	CG	Gene Title	Process/Function	*w^1118^* hypoxia vs. *sima* hypoxia	*w^1118^* normoxia vs. *w^1118^* hypoxia
1639737_at	CG34330	—	—	−67.60	4.82
AFFX-Dm-U46493-1_s_at	—	—	—	−22.19	2.47
1625173_s_at	CG11652	dDPH1	diphthamide synthesis	−16.82	10.91
1626857_at	CG4408	—	carboxypeptidase	−11.90	−2.93
1627135_at	CG4608	branchless	FGF receptor	−9.51	10.87
1637758_at	CG7737	—	spermine oxidase	−7.93	8.56
1638797_a_at	CG31543	Fatiga	HIF prolyl hydroxylase	−7.91	7.70
1637182_at	CG9503	—	choline dehydrogenase	−6.85	2.51
1624497_at	CG2676	—	—	−6.44	4.28
1634786_at	CG7106	lectin-28C	mannose receptor	−6.01	1.80
1628705_at	CG31022	PH4alphaEB	prolyl hydroxylase	−5.62	3.08
1636482_at	CG14005	—	—	−5.34	5.69
1639555_at	CG17724	—	—	−4.66	4.23
1629753_at	CG3340	Kruppel	transcriptional repression	−4.47	−1.76
1632203_at	CG31706	—	—	−4.26	6.16
1628428_at	CG12389	dFPP	geranyltranstransferase	−4.25	3.79
1635558_s_at	CG17724//CG32904	—//sequoia	—	−4.19	4.31
1627525_a_at	CG1333	Ero1L	protein disulfide isomerase	−3.79	5.91
1636145_at	CG7219	Serpin 28D	serine-type endopeptidase inhibitor	−3.78	3.87
1626844_at	CG5748	HSF1	transcriptional activator	−3.54	2.98

Top 20 HIF-Independent Hypoxia Response Genes				*sima* normoxia vs. *sima* hypoxia	*w^1118^* normoxia vs. *w^1118^* hypoxia
1631533_at	CG10240	Cyp6a22	E-class cytochrome P450	7.82	5.38
1638182_at	CG5999	—	UDP-glucuronosyltransferase	7.49	5.89
1635227_at	CG10160	ImpL3	lactate dehydrogenase	7.38	14.16
1626324_at	CG9964	Cyp309a1	E-class cytochrome P450	6.48	3.57
1628758_at	CG1774	dACOT	acyl-CoA thioesterase	6.36	4.39
1632021_at	CG10245	Cyp6a20	E-class cytochrome P450	6.30	2.79
1629061_s_at	CG32041	Hsp22//Hsp67Bb	response to stress	5.83	6.01
1628052_at	CG10241	Cyp6a17	E-class cytochrome P450	5.20	5.67
1632343_at	CG3017	Alas	5-aminolevulinate synthase	5.07	3.82
1631637_a_at	CG11567	Cpr	NADPH-cytochrome P450 reductase	4.91	4.36
1639495_at	CG4485	Cyp9b1	E-class cytochrome P450	4.80	5.44
1628660_at	CG7130	Hsp40	dnaJ homolog	4.18	3.05
1624156_at	CG18578	Ugt86Da	UDP-glucuronosyltransferase	4.02	3.06
1632639_at	CG13941	Arc2	synaptic plasticity	3.94	3.44
1631611_at	CG33983	obstructor-H	chitin-binding	3.94	2.39
1641169_s_at	CG11050	—	phosphoric diester hydrolase	3.71	3.68
1634072_s_at	CG4311	Hmgs	HMG-CoA synthase	3.68	2.53
1625134_at	CG10873	p53	tumor suppressor	3.57	2.36
1630885_at	CG12534	Alr	flavin-linked sulfhydryl oxidase	3.52	1.97
1628657_at	CG17534	GstE9	glutathione S transferase	3.38	3.89

For HIF-dependent genes (taken from the top 20 down-regulated transcripts in the HD H-genes set), transcripts are sorted according to normalized microarray values obtained when comparing *w^1118^* hypoxia samples with *sima* hypoxia samples. For the HIF-independent genes (taken from the top 20 up-regulated HI H-genes set), transcripts are sorted according to the normalized microarray values obtained when comparing *sima* normoxia with *sima* hypoxia samples. For comparative purposes, the respective hypoxic changes observed in *w^1118^* control animals are reported in the last column. Additionally, the first four columns show the Affymetrix probe set ID, CG number, gene title, and the putative process/function of the encoded protein.

**Figure 2:**
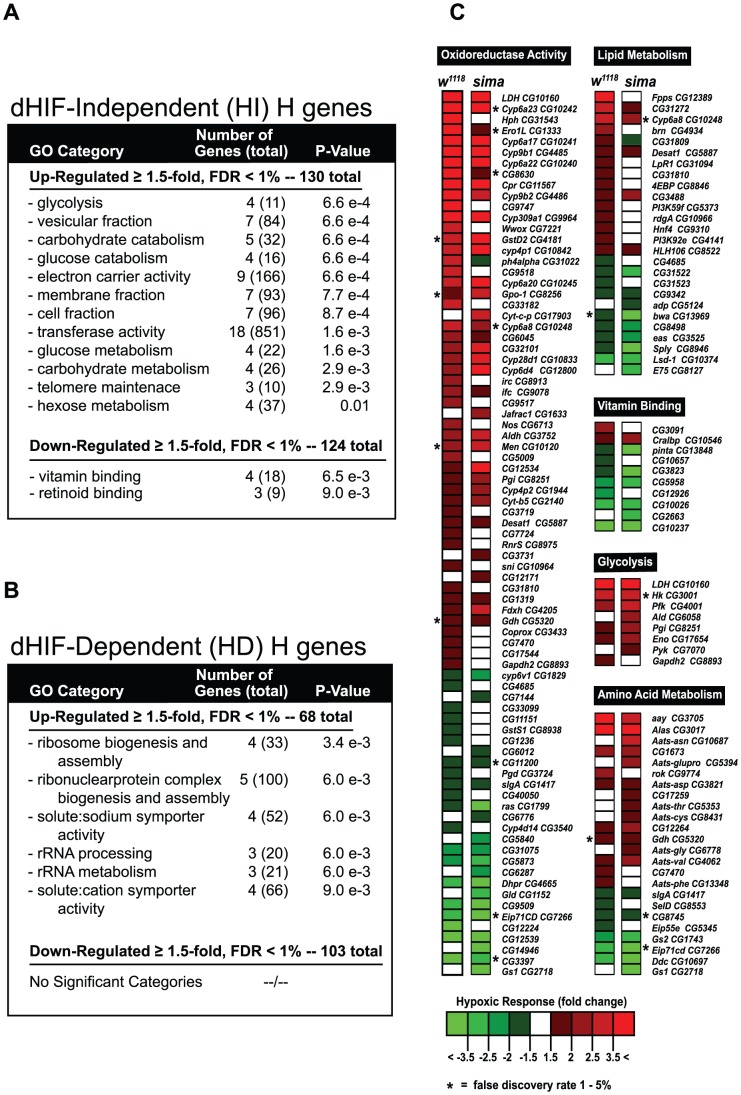
HIF-dependent and HIF-independent hypoxic response genes. (A–B) Gene ontology (GO) analysis was performed on HIF-independent (HI) and HIF-dependent (HD) gene sets that were derived from microarray analysis of H- or N-treated control (*w^1118^*) animals or *sima* mutants collected at the partial clear-gut late-L3 time. See Figure S1 for the analysis scheme. GO categories are listed in order of statistical significance. The numbers of H-regulated genes affected are shown along with the total number of genes in each category. All transcripts are up- or downregulated at least 1.5-fold and have a false discovery rate (FDR) of <1%. (C) A heat map was created to illustrate the similarity or dissimilarity of hypoxic responses for major GO categories impacted by H-treatment in control animals and *sima* mutants. Red (upregulated), green (downregulated), or white (no significant change) values represent hypoxic responses observed in the backgrounds indicated. For this analysis, FDR stringency was <1%, unless otherwise noted with * for a particular genotype, where a relaxed gate was used (1–5%).

Gene ontology (GO) analysis [44] was performed on the hypoxia genes sets (Figure 2A and 2B). Notably, the HI genes set, and not the HD genes set, contain glycolytic transcripts that are upregulated in hypoxia, which was the single most statistically-impacted process in either the HD or the HI sets (Figure 2A). Instead of glycolytic genes, significant GO categories were identified for HD genes involved in translational control and RNA processing (Figure 2B). However, among the HD H-regulated transcripts are *fatiga* and *dVHL*. This suggests that dHIFa participates in a feedback regulatory loop that attenuates its own activity.

Ontology-focused heat maps were generated to compare hypoxic transcriptional responsiveness. In addition to glycolytic genes, comparisons were made for other metabolic categories where GO significance was identified, including oxidoreductase activity, lipid metabolism, vitamin binding, and amino acid metabolism (Figure 2C). With the exception of lipid metabolic genes, when hypoxic responsiveness is seen (up- or downregulated) in the control background, the majority of genes also respond in-kind in the *sima* background, and usually with a similar fold-change increase. These results suggest that HIF-independent, H-sensitive mechanisms account for a large percentage of the hypoxic response.

### Temporal-dependent hypoxic responses

The unexpected breadth of contribution of the HI pathway in the hypoxic response led us to reconsider our initial observations made in Figure 1, where *fatiga* displayed a similar response profile across each of the times assayed, and *LDH* and *Pfk* displayed a hypoxic response at only the late-larval time. Indeed, *fatiga* is a HD gene, whereas *LDH* and *Pfk* are HI genes (Table S1). Were *LDH* and *Pfk* unresponsive at earlier developmental times because the HI pathway was not active until just prior to metamorphic onset? To address this question, we collected RNA from control animals and *sima* mutants staged at times that spanned development. In all, twelve samples were gathered: 4 embryonic times (0–6 hrs AEL [*w^1118^* background only], 6–12 hrs AEL, 12–18 hrs AEL, and 18–24 hrs AEL); 4 larval times (mid-L1, mid-L2, mid-L3, and −4 hr RTP); 3 metamorphic times (0 hr RTP, +12 hr RTP, +72 hr RTP); and 1 adult time (1 day-old males). qRT-PCR was used to assess H responses of 13 select genes that displayed varying levels of H-sensitive expression. Of those genes analyzed: five were classified as HD genes – *fatiga, spermine oxidase* (*CG7737*), *sequoia* (*CG17724*), *branchless* (*CG4608*), and *Peroxiredoxin 2540-2* (*Prx2540*, *CG*11765); seven were classified as HI genes – *LDH, Pfk, NMNAT* (*CG13645*), *Alas* (*CG3017*), *Cyp9b1* (*CG4485*), *Cyp6a17* (*CG10241*), and *Cyp6a22* (*CG10240*); and one was highly affected by H, but did not meet the stringency requirements for H set inclusion – *CG31769*, which had a largely HIF-dependent expression profile in late-L3.

**Figure 3:**
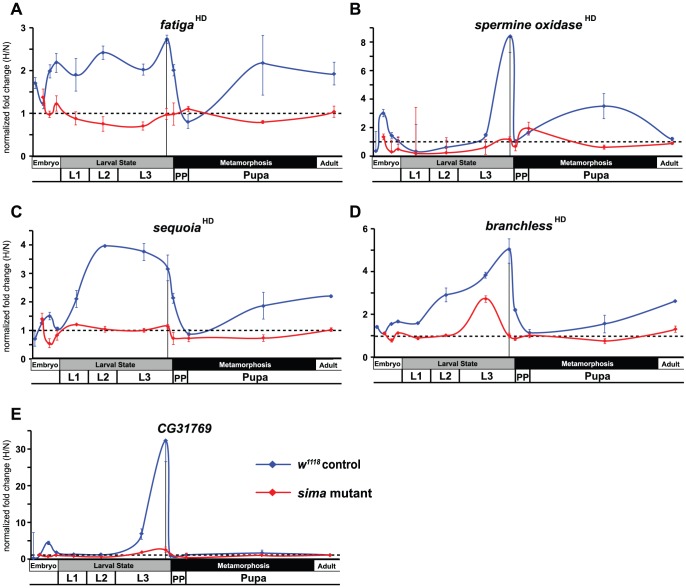
Temporal expression of HIF-dependent hypoxic response genes. (A–E) Developmental hypoxic response profiles from qRT-PCR analyses are shown for transcripts that display HIF-dependent (A – *fatiga*, B – *spermine oxidase*, C – *sequoia*, D – *branchless*) or largely HIF-dependent expression (E – *CG31769*). Control (*w^1118^*) animals or *sima* mutants were challenged for 6-hrs with 4% O_2_. Animals were challenged at: 0–6 hr after egg laying (AEL) (not for mutant), 6–12 hr AEL, 12–18 hr AEL, 18–24 hr AEL, mid-L1, mid-L2, mid-L3, −10–−4 hr relative to pupariation (RTP) L3, 0 hr RTP, +12 hr RTP, +72 hr RTP, and 1-day old males. All values are from experiments performed in triplicate from pools of biological replicates. Values are normalized to *rp49* expression and are reported as the relative fold-change of H/N. The dotted line represents no net change in response, or a value of 1.0. The vertical black line in late-L3 is when microarray analysis was performed. Error bars are the SEM. Separate normoxic and hypoxic traces for each transcript surveyed are shown in Figure S3.

**Figure 4:**
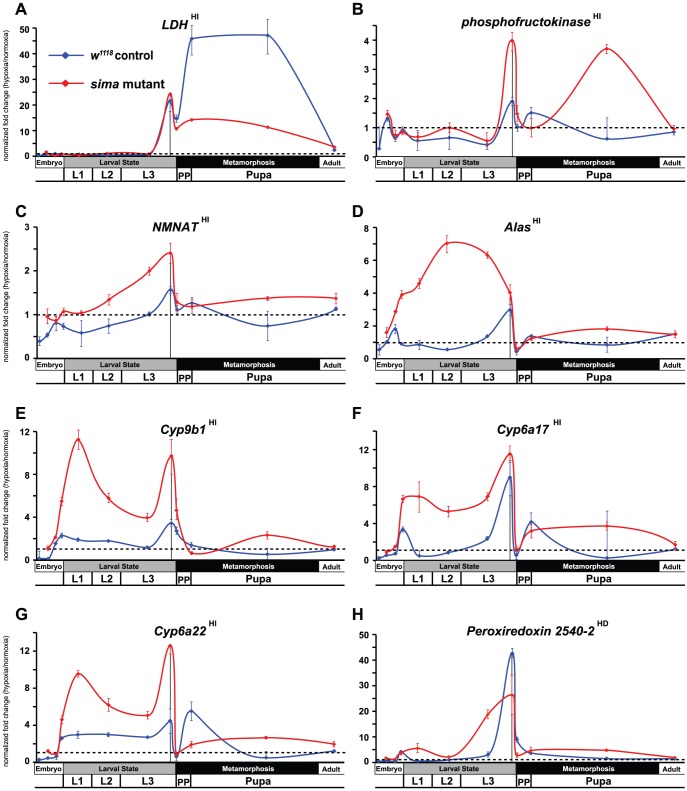
Temporal expression of HIF-independent hypoxic response genes. (A–H) Developmental hypoxic response profiles from qRT-PCR analyses are shown for transcripts that display HIF-independent (A – *LDH*, B – *phosphofructokinase*, C – *NMNAT*, D – *Alas*, E – *Cyp9b1*, F – *Cyp6a17*, G – *Cyp6a22*) or largely HIF-independent expression (H – *Peroxiredoxin 2540-2* – we note that *Prx2540-2* displays HD expression for probe set 1631628_s_at in the microarray and is thus classified as such; however, a second, non-overlapping probe set, 1633471_at, which is much more robustly induced in H appears to be from HI action, although its variability is too great to classify in this manner (Table S1). Our qPCR primer set favors HI expression.) Control (*w^1118^*) animals or *sima* mutants were challenged for 6-hrs with 4% O_2_. Developmental challenge times are identical to those from Figure 3. All values are from experiments performed in triplicate from pools of biological replicates. Values are normalized to *rp49* expression and are reported as the relative fold-change of H/N. The dotted line represents no net change in response, or a value of 1.0. The vertical black line in late-L3 is when microarray analysis was performed. Error bars are the SEM. Separate normoxic and hypoxic traces for each transcript surveyed is shown in Figure S4.

Several patterns emerged from the developmental analysis. First, hypoxic transcriptional induction is most evident at the late-L3 time for each gene assayed. Second, without exception, HD and HI genes display marked drops in H responsiveness just after metamorphic onset. In most cases, H responsiveness is eliminated during the hours surrounding head eversion, which is the initiation of the pupal phase. Among the transcripts examined, HD genes were not induced in hypoxia in a *sima* background at any developmental time, with the notable exception of a single point in mid-L3 for *branchless* (Figure 3D). Finally, in the absence of dHIFa, HI genes tend to be hyper-responsive to H challenge throughout development – this was true for all genes examined except *LDH* (Figure 4A). The *LDH* profile was unique amongst those assayed, in that late-L3 expression was HI, while pupal expression appears to be dominated by HD expression. The super-activation of *LDH* during the pupal phase in the *w^1118^* background (vs. *sima*) suggests that both the HD and HI pathways are capable of converging simultaneously at the same locus to contribute to its overall expression. Collectively, these developmental expression data indicate that hypoxic responses are comprised of a patchwork of HD and HI activities throughout life-stage progression.

### *sima* mutants are metabolically deranged in normoxia and are unable to mobilize glycogen in hypoxia

**Figure 5:**
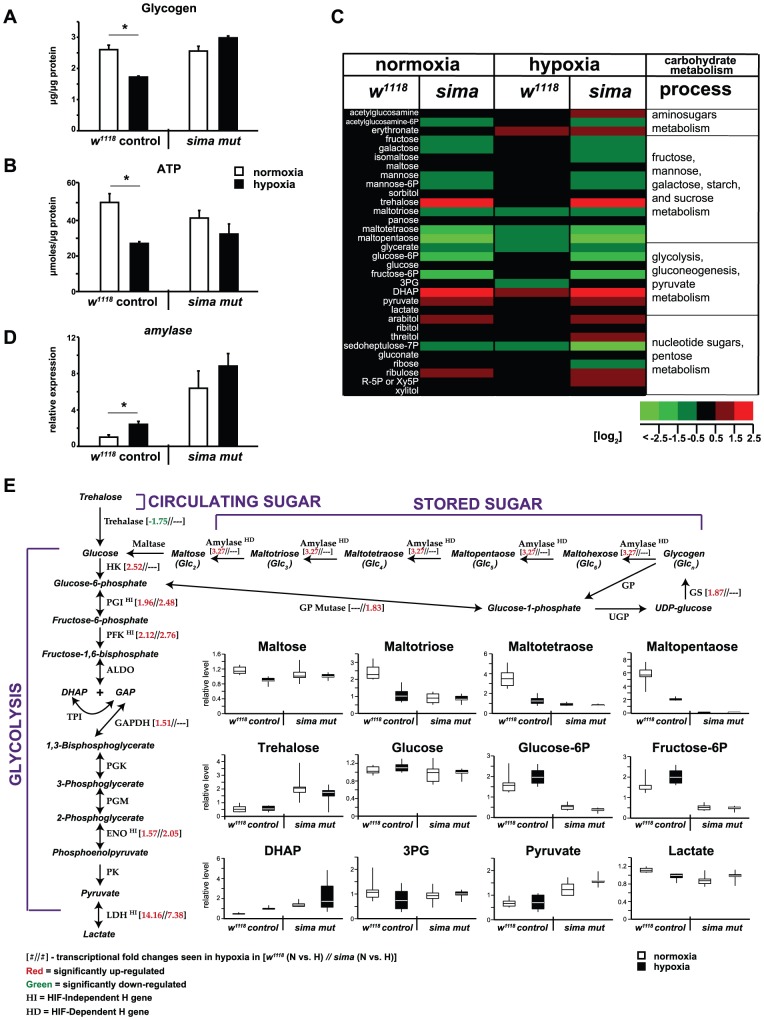
HIF-dependent effects on carbohydrate catabolism. (A) Unlike the *w^1118^* response, *sima* mutants are not able to mobilize glycogen stores in response to 6-hr H-treatment in late-L3. (B) ATP levels are significantly depleted upon H-challenge by *w^1118^* animals. Although sima animals showed a similar trend, the decrease observed was not significant. Levels of glycogen and ATP are normalized to total protein. (C) Shown is a metabolic heat map of individual metabolites measured by GC/MS or LC/MS from late-L3 *w^1118^* animals or *sima* mutants subject to N (left two columns) or H (right two columns). Six replicates were measured for each treatment group. Each replicate has ∼250 independently collected and pooled animals. Metabolite levels are expressed as log_2_ transformations of the average values, which are plotted relative to the normoxic level obtained in the WT background. The data are normalized to total protein content. Red indicates an elevated metabolite level, green indicates a diminished level, and black no/little change. See Table S2 for further information and statistics. (D) qRT-PCR analysis showing *amylase* expression increases in hypoxia in control animals. Although *sima* mutants have an elevated constitutive level of *amylase* expression, they do not induce expression in hypoxia. (E) Integrated snapshot of the transcriptional and metabolic response to hypoxia at 6-hr. Shown, are the metabolites and enzymes of stored sugar and circulating sugar as they feed into glycolysis. When significant H-induced changes were noted (fold-change >1.5, FDR <1%) for transcripts in microarray experiments, those changes are noted in brackets next to the enzyme names. HD or HI status, as classified in Table S1 is also noted. Metabolite levels from mass spec analysis are additionally shown as box and whisker plots. The upper and lower boundaries of the box note the upper and lower quartile values, while the ends of the vertical line indicate the maximum and minimum values. The average value is the noted by the horizontal line within the box. Error bars are the SEM. * = *p*-value<0.05.

The observation that glycolytic transcripts are effectively upregulated in *sima* mutants challenged with hypoxia raised the question of how metabolism was affected under these conditions. As before, we concentrated on the late-L3 time because of its particularly robust transcriptional response to H-treatment. We found that glycogen was significantly depleted by control *w^1118^* animals in H, in addition to a near 50% reduction in the level of ATP (Figure 5A and 5B).

We tested for additional HIF-dependent metabolic defects in carbohydrate catabolism using mass spectrometry tied to gas and/or liquid chromatography (GC/MS, LC/MS). Extracts were prepared from animals subjected to N- and H-treatments and 32 carbohydrate metabolites were measured (Table S2). The metabolites correspond to four broad categories: 1) aminosugar metabolism; 2) fructose, mannose, galactose, starch, and sucrose metabolism; 3) glycolysis, gluconeogenesis, and pyruvate metabolism; and 4) nucleotide sugars and pentose metabolism (Figure 5C).

We found that the control response to hypoxia is characterized by a remarkable level of metabolic stability for carbohydrate catabolites (third column in Figure 5C). Among those compounds that do display significant H-induced depletions are oligomeric forms of glucose (maltose, maltotriose, maltotetraose, and maltopentaose), which are catabolic products from glycogen and starch breakdown (Figure 5C and 5E). These sugars feed into the glycolytic cascade by replenishing glucose. They are successively depleted in H the larger they are, and their reductions are consistent with a depletion of total glycogen seen in the *w^1118^* response (Figure 5A), as well as the HIF-dependent upregulation of *amylase* in H in the same background (Figure 5D).

In contrast to the effects that H-treatment has on *w^1118^* animals, *sima* mutants cannot deplete glycogen in H (Figure 5A). Instead, they adopt a profile for the maltose oligomers in normoxia that resembles the hypoxia-mobilized profile in control animals (Figure 5C and 5E). This is likely a combination of two factors – the *sima* mutant's inability to effectively upregulate *amylase* in H and its constitutively elevated expression profile for *amylase* in normoxia that is greater than *w^1118^* expression in hypoxia (Figure 5D, Table S1).

Curiously, despite the clear transcriptional switch toward glycolytic energy production at late-L3, lactate levels remained unchanged for either genotype in H (Figure 5C). This failure to generate lactate in hypoxia is a stage-specific block. We independently performed lactate measurements by an enzymatic assay to confirm the late-L3 findings made by GC/MS. Indeed, we find that mid-L1 larvae and young adults from either the *w^1118^* or *sima* backgrounds produce lactate in hypoxia, but not late-L3 larvae (Figure S5A–S5C). Notably, early larval and young adult *sima* mutants exhibit an exacerbated hyperlactatemic phenotype when subject to hypoxia (Figure S5A, S5C) – this does not happen to late-L3 animals.

Additionally, though the transcriptional H response profile was largely normal for glycolytic genes in the *sima* mutant, profound depletions were still observed for glucose-6-phosphate and fructose-6-phosphate in H (Figure 5C and 5E). This is because the normoxic levels for these compounds, rather than H-induced changes, dominate their metabolism. We also observed a HIF-dependent increase for pyruvate in N and H. This is consistent with findings in *HIF-1a^−/−^* MEFs, which maintain higher levels of ATP in hypoxia than *WT* MEFs do in normoxia [45]. Finally, the elevated level of Ru5P:Xu5P and ribulose, coupled with the depleted levels of S7P, reveal that *sima* mutants display a clear split in the oxidative (NADPH-generating) and non-oxidative phases of the pentose phosphate pathway in normoxia, which is exacerbated by H-treatment.

### dERR binds to dHIFa

The only factor known to transcriptionally regulate glycolytic transcripts in *Drosophila* is dERR [32]. Our lab identified this orphan nuclear receptor as a potential factor that may participate in hypoxic signaling when the dERR ligand-binding domain (LBD) was used to repeatedly isolate *sima* clones in a large-scale yeast two-hybrid screen. Of the 20 positive clones recovered in the screen, seven encoded different C-terminal fragments of dHIFa. These findings are consistent with a previous report that demonstrated HIF/ERR interactions between the *Drosophila* proteins and their mammalian homologs [39]; however, there are two important aspects about HIF/ERR complexes that we note differentiate the fly and mammalian complexes. First, we find that the dERR DBD is dispensable for interaction with dHIFa, whereas the Ao report showed that interaction occurs between the mammalian ERR DBD and the HIF-1a/b heterodimer. Second, unlike in mammals [39], HIF-1b (tango) is not required for dERR association with dHIFa in *Drosophila* – tango was not present in the screen. On this aspect, our findings are consistent with the findings made by Ao et al. Their two-hybrid screen of *Drosophila* components also did not have a HIF-1b [39].

**Figure 6:**
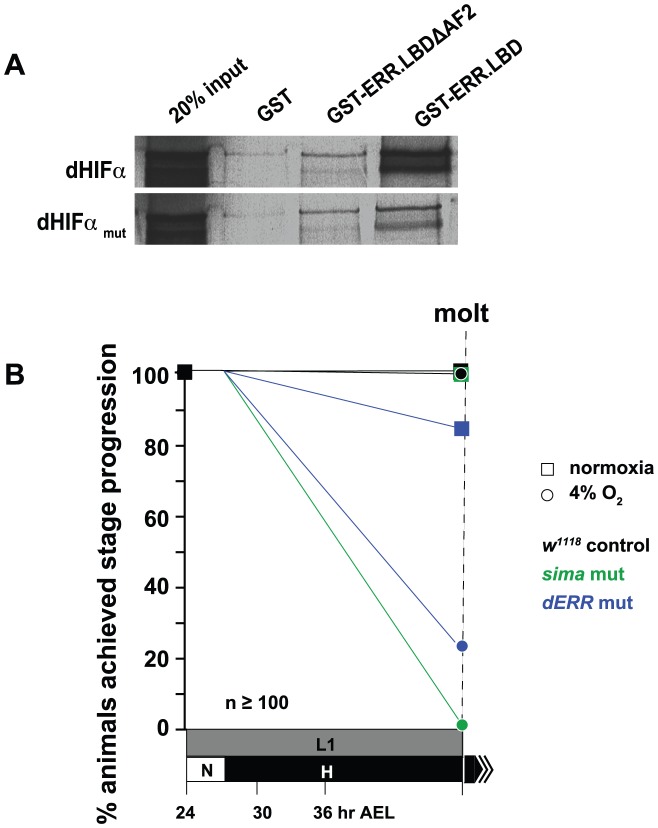
dERR binds to dHIFa and is essential for hypoxic survival. (A) GST-pulldown experiment showing GST-fused dERR LBD association with full-length dHIFa, which is diminished when the final 11 amino acids of the LBD are deleted (ΔAF-2). Similarly, when the LXXLL motif in dHIFa is mutated, binding with the ERR LBD and ΔAF-2 proteins is lessened, but not eliminated when compared to GST alone. (B) *dERR* mutant animals are sensitive to H exposure and fail to successfully progress to the molt when challenged with 4% O_2_. Shown also are the results of *w^1118^* animals and *sima* mutants. The L1 stage takes ∼24 hrs to progress through in N at 25°C, but the allotted time was extended to 48 hrs to account for developmental delays that are caused by H treatment. For more details and additional data, see Table S3.

We validated our two-hybrid screen findings by performing a GST-pulldown with GST-fused dERR LBD protein with full-length dHIFa, which confirmed a robust interaction (Figure 6A). The C-terminal AF-2 helix of nuclear receptors often mediates interaction with transcriptional coregulators through an LXXLL motif that is found on the interacting protein [46]. A single such sequence resides within dHIFa, at amino acids 1289–1293 (LKNLL). When the last two leucines of this sequence were mutated to alanine and/or when the last 12 amino acids of the dERR LBD were deleted, spanning the AF-2 helix (479–491), the interaction between the proteins was severely reduced, but not eliminated (Figure 6A). These data indicate that the dERR AF-2 helix mediates a docking point with the dHIFa LXXLL motif, but that at least one additional point of contact is maintained between dERR and dHIFa.

### *dERR* mutants are hypoxia-sensitive

We have recently shown that the orphan nuclear receptor dERR is essential for triggering the pro-growth glycolytic program during *Drosophila* development [32]. Without the dERR-initiated metabolic switch, development cannot successfully proceed. Many of the same metabolic genes that exhibit H-sensitive regulation are also misregulated in the *dERR* mutant. If dERR is important in the hypoxic response, as suggested by its association with dHIFa, then the mutants should be sensitive to H-treatment. To test this, we challenged *dERR* mutants and compared their H-sensitivity with *sima* mutants and control animals. Indeed, 24–30 hr AEL L1 larvae challenged with constant hypoxia resulted in *sima* mutant lethality (Figure 6B). The *dERR* mutants were also H-sensitive, but not to the same extent as *sima* embryos. Nevertheless, dERR is critical, less than 25% of animals survived as compared to 97% survival for the *w^1118^* background. These data indicate that dERR is essential for hypoxic adaptation.

### dERR is essential for HIF- and non-HIF-dependent responses

**Figure 7:**
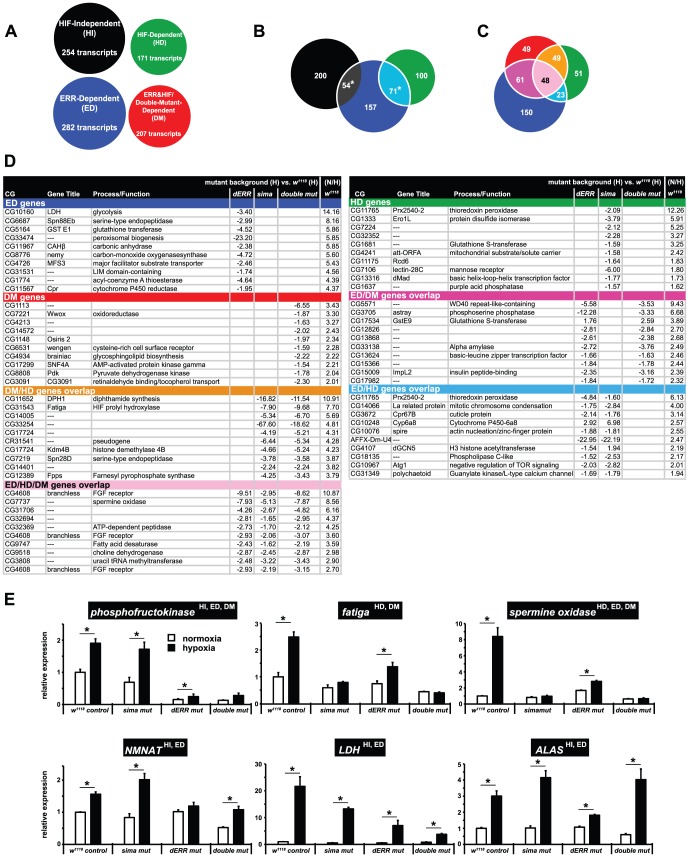
The influence of dERR and dHIFa on hypoxic transcripts. (A) HIF-independent (HI), HIF-dependent (HD), ERR-dependent (ED), and ERR&HIF-dependent (DM) gene sets identified by microarray schemes outlined in Figures S1 and S2. Circles are scaled to size by number of transcripts in each set. (B) A Venn diagram demonstrating the overlap of the HI/HD/ED H-genes sets. Note, HI and HD genes sets are, by definition, mutually exclusive. The asterisks indicate that the overlap is significant (*p*-value<0.05), as determined by hypergeometric probability. (C) A Venn diagram demonstrating the overlap of the HD/ED/DM H-genes sets. qRT-PCR analysis of hypoxia-regulated genes falling into specific Venn overlaps, as indicated by arrows. (D) The top ten affected transcripts, as assessed by the H-responses measured in the control background, for each of the seven Venn categories shown in Figure 7C. Hypoxic expression for each transcript in the different mutant backgrounds (compared to *w^1118^*) is reported as fold-change difference. Additionally shown is the N/H ratio obtained for *w^1118^* animals. (E) Normoxic and hypoxic expression of each of the six genes (*Pfk, fatiga, spermine oxidase, NMNAT, LDH, ALAS*) was determined using RNA collected from animals of the indicated genotypes at late-L3. Samples were collected in triplicate and are independent from those used in the microarrays. Values are normalized to *rp49* expression and are reported relative to the value obtained for *w^1118^* in normoxia.

Using the same analytic framework that was used to assess *sima* involvement in the late-L3 larval hypoxic response, we collected RNA samples from *dERR* mutants and *dERR,sima* double-mutants for microarray analysis to determine how loss of ERR alone or ERR and dHIFa together would affect hypoxic responses (Figure S2). Through these analyses, we identified 282 dERR-dependent (ED) transcripts and 207 double-mutant-dependent (DM) transcripts whose expression changed in hypoxia (Figure 7A, Table S1). The ED and DM H-genes sets encompass a variety of highly significant GO categories, including H-induced kinases and transferases that specifically require dERR, and a host of nucleolar and RNA processing transcripts that are coordinately upregulated in hypoxia due to the lack of both dERR and dHIFa (Figure S6A, S6B, Table S1).

Venn analysis was used to assess the similarity of the independently derived H-gene sets (HI, HD, ED, DM). The overlapping pattern of the ED genes set with the mutually exclusive HI and HD sets demonstrates that dERR significantly affects both HIF-dependent H-genes (71 transcripts) and HIF-independent genes (54 transcripts) (Figure 7B). Interestingly, among the overlap between the HI and ED genes sets are all the glycolytic transcripts that are upregulated in hypoxia. These data reinforce our earlier findings that demonstrate that at metamorphic onset, dHIF is not part of the hypoxic-induced glycolytic shift. They also suggest that a portion of the HI response it attributable to dERR.

Given that dERR can interact with dHIFa, and that it can impact hypoxic transcription independent of HIF, we anticipated that the DM H-genes set would significantly overlap the ED and HD genes sets. Indeed, this is the case – as shown by Venn analysis, the DM set has more overlap with the HD and ED sets than not (Figure 7C). A listing of the top hypoxia-sensitive transcripts in the various Venn overlapping regions can be found in Figure 7D.

To verify that loss of dERR and/or dHIFa selectively eliminates/diminishes hypoxic induction, RNA samples were independently collected from control animals, *sima* mutants, *dERR* mutants, and double-mutants (Figure 7D). Six genes were chosen for further analysis by qRT-PCR. The results demonstrate that the factor-dependent classification we employed for hypoxic responsiveness is accurate. For example, *Pfk* is classified as HI, ED, and DM, indicating that hypoxic regulation should be affected in the double-mutant and the *dERR* mutant backgrounds, but unaffected in the *sima* mutant – this is the pattern that is observed (Figure 7E). Similar trends also held true for *fatiga* and *spermine oxidase*, which were expected, respectively, to only respond in the *dERR* mutant background, or not in any of the three mutant lines. With the exception of a modest H-induction in the *dERR* mutant for *spermine oxidase* the responses were true. Hypoxic responses for *NMNAT*, *LDH*, and *ALAS* were all expected to display the same pattern; which is that only in the *dERR* background will H-responsiveness be significantly reduced/eliminated. Responses were, by-and-large, as expected, except for the significant H-induction of *LDH* in the in *dERR* mutants.

These data indicate that dERR and dHIFa have a different activity profile when in the presence of the other, than either protein has by itself, and suggest that promoter-specific actions of different HIF and/or ERR complexes drive a large percent of hypoxic responses at metamorphic onset. In certain cases, loss of one factor does not influence the other's response, as with loss of dHIFa for the dERR-mediated *Pfk* response (Figure 7E). In other cases, loss of either dHIFa or dERR renders the H-response incomplete, such as occurs with *spermine oxidase*. And, still in other cases, loss of one factor is more detrimental for H-induction than is loss of both, as with *ALAS*. Responses of this type appear to suggest that, at certain loci, dHIFa acts as a negative regulator of hypoxic transcription in the absence of dERR but not in its presence.

## Discussion

Our results underscore the complexities of adaptive responses in hypoxia, which are life-stage specific and controlled by multiple H-sensitive pathways. Although our data confirm that HIF is a major transcriptional driver of hypoxic responses, we also define distinct HIF-independent responses. These data raise new questions about dHIFa collaboration and challenge the notion that the HIF complex has little or no normoxic role. In addition, we show that a significant fraction of HIF-independent pathways can be attributed to the ERR nuclear receptor.

Among the HIF-independent genes were numerous glycolytic transcripts that are well-known responders to hypoxia [26], [31], [40]. The fact that these genes are as effectively upregulated in *sima* mutants as they are in a control response was surprising, particularly considering the known role of HIF-1a in this process [47]. We find that dERR is the overriding factor that mediates hypoxic upregulation of glycolytic genes (*Pgi*, *Pfk*, *GAPDH2*, *enolase*) just prior to metamorphic onset.

Our findings, however, do not exclude dHIFa contribution in hypoxic expression of HI genes at other developmental times. The super-induction of *LDH* during metamorphosis in *w^1118^* animals versus *sima* mutants is consistent with this scenario (Figure 4A). These temporal- and context-specific differences may explain the wide variability in hypoxic responses that have been seen between cell-types [16], [48], despite the ubiquitous presence of the HIF pathway. Furthermore, they may account for discrepancies between our data collected on *Drosophila* and reports on mammalian systems. For example, *LDH* is a HIF-independent hypoxia-regulated gene in late-L3 animals. However, loss of dERR has a greater effect on the diminution of hypoxic induction at this developmental time than does loss of dHIFa (Figure 7C). But, this effect is short-lived, because just hours later, when the larva transitions into a pupa, dHIFa appears to work in combination with a non-HIF pathway to elicit hypoxic responsiveness (Figure 4A). This combinatorial response during *Drosophila* metamorphosis is consistent with vertebrate studies that show *LDH* expression is the product of HIF-1 action that also requires the presence a cAMP response element for full hypoxic induction [47], [49]. Thus, in addition to different pools of potential coregulatory molecules that may significantly alter HIF-dependent transcription, entirely different transcriptional pathways, with their own triggers of hypoxic induction, refine the H response. Given the right spatiotemporal setting, HIF-independent pathways may displace (or substitute for) the HIF pathway altogether, a result that is consistent with our data. Further support of this idea is evident in the expression of Alas2, the rate-limiting enzyme for heme production. *Alas2* has been identified as a HIF-dependent and a HIF-independent hypoxia-regulated gene in mammals [50]–[52]. In our hands, *ALAS* is H-responsive, and displays HIF-independent and ERR-dependent upregulation, which may be subject to dHIFa negative regulation in dERR's absence (Figure 7E).

The dynamic patterns of temporal expression of HI and HD genes raise the fundamental question of how hypoxic responses are regulated through development and into the adult. Low-oxygen responses are not one-size-fits-all programs that mitigate oxidative damage and metabolic imbalance; they must be coordinated with developmental progression and metabolic state. In particular, late-L3 wandering larvae exhibit a hypersensitive transcriptional response to hypoxia for HIF-independent/ERR-dependent glycolytic genes. This includes a robust *LDH* induction (Figure 4A). Paradoxically, however, late-L3 larvae do not produce lactate in the 6-hr hypoxic challenge (Figure 5C, 5E, and Figure S5). In contrast, at other developmental times (L1, adult), animals correspondingly produce lactate in hypoxia, even though they remain transcriptionally incompetent to induce *LDH* transcript (Figure S5 and Figure 4A). We speculate that the atypical transcriptional and metabolic hypoxic profiles of the late-L3 larva are a product of its developmentally programmed energetic state, which at this time is transitioning from low to high efficiency (see the dramatic decrease of *Pfk* expression in late larvae in Figure S4). Just prior to the wandering L3 time, larvae are prolifically growing, and in a state of metabolism that is fueled by aerobic glycolysis – this metabolic program is ERR-dependent [32]. Just after this developmental time, larvae initiate metamorphosis, which will impose 5 days of developmentally forced starvation. During this lipid-driven phase [53], metabolism is characterized by high efficiency OXPHOS.

In contrast to the switch-like hypoxic expression of HIF-independent glycolytic transcripts, the HIF prolyl hydroxylase *fatiga* displays relatively uniform expression throughout development (Figure 3A and Figure S3), suggesting that regulation of the HIF pathway, by HIF itself, is equally important at all times for the animal. Such disparities in induction are only understood in context. While our studies here provide a framework with which to view H responses, they indicate that further developmental analysis is needed to more fully appreciate hypoxic response pathways and the mechanisms that specifically support their activities.

Although we have emphasized the transcriptional and metabolic impacts of hypoxia on carbohydrate catabolism, the breadth of our data sets indicate that many important hypoxia-induced changes are thus far unappreciated and await further investigation. What is the significance, for instance, of the greater than 10-fold increase of HIF-dependent expression of *dDPH-1* (*CG11652*) in hypoxia (Table 1)? DPH-1 is a tumor suppressor that is responsible for the first step of the unique protein modification that occurs on elongation factor 2 (eEF2), which converts a histidine residue to diphthamide. This residue is the target of diphtheria toxin that can shut down protein synthesis through ADP-ribosylation. Although diphthamide formation is conserved from archaea to human, its significance on cellular function is not clear, as it is dispensable for protein elongation [54]. However, it has been implicated in translational fidelity [55] and is likely an asset under stress [56]. GO analysis performed on HD H-regulated genes indicate that dHIFa is important in replenishing select protein translation/RNA processing transcripts. From this perspective, *DPH-1* induction by dHIFa may be indicative of a regulatory role of hypoxic translation for HIFs. Such a role would be consistent with a recent report from mammals that demonstrates a HIF-2a-dependent association with ribosomal/translational control proteins and the selective hypoxic translation of transcripts containing an RNA hypoxic response element in the 3′UTR via a mechanism involving eIF4E2 [57].

Our analysis of carbohydrate catabolism identifies amylase-mediated breakdown of glycogen as the fuel of first resort in hypoxia (Figure 5). This catabolic pathway feeds into glycolysis and supplies needed glucose for increased glycolytic flux, obviating the need to draw on circulating sugar in the form of trehalose, which did not change in the 6-hr challenge. The strategy of glycogen mobilization allows animals to maintain a remarkably stable profile for a wide variety of carbohydrate catabolites.

Trehalose levels are substantially elevated in *sima* mutants, regardless of oxygen status (Figure 5C and 5E). These data may indicate a role for dHIFa in the insulin receptor pathway. Numerous studies demonstrate that trehalose levels are altered by genetic disruptions of the insulin-signaling components [58]–[61]. Alternatively, elevated trehalose levels may be the result of constitutively high expression of *amylase* (Figure 5D). Although the increased *amylase* expression does not translate into a depleted level of glycogen in the *sima* mutant (Figure 5A), it is conceivable that increased glycogen deposition compensates for increased glycogenolysis.

It is important to note that post-transcriptional control mechanisms are well known to impact glycolytic enzymes. Although we did not document them, we consider such influences on hypoxic glycolytic flux likely to have genotype-specific effects.

*sima* mutants do not mobilize glycogen in hypoxia, but they are able to initiate H-induced changes for other carbohydrates. This is the case for the glycolytic intermediate DHAP, which more than doubles in a control hypoxic response and significantly accumulates in mutants (Figure 5C, 5E). These findings are consistent with appropriate transcriptional responses we noted for glycolytic transcripts in *sima* animals, which are upregulated in hypoxia by dERR, not dHIFa. The results for glycogen notwithstanding, it is the widespread derangement of normoxic set points for metabolites that characterizes the metabolic incompetency of the *sima* mutant. Our data indicate that dHIFa has it greatest impact on metabolism in the unchallenged normoxic state, rather than in hypoxia.

The mechanism whereby dERR participates in hypoxic responses needs to be explored further. We identified dERR as a potential player in hypoxic responses through its association with dHIFa (Figure 6A), suggesting that it acts in a collaborative role with the HIF complex through direct recruitment to HREs. This model was favored by the Ao et al. report for ERR participation in hypoxic responses in vertebrates [39]. Additionally, dERR may recruit dHIFa to ERR-specific response elements to facilitate H responses. Another possibility is that dERR actively regulates hypoxic transcription without dHIFa at all; or, in parallel to the actions of dHIFa, which may occur independently, yet simultaneously. Each of these scenarios is consistent with hypoxic expression analysis that we performed to generate HD, HI, ED, and DM gene sets. Moreover, in the presence of dERR, dHIFa may act as a negative regulator of hypoxic responses at select hypoxia-regulated sites (Figure 7E, *NMNAT*, *ALAS*). Of further interest also, will be the identification of the triggers for ERR participation in hypoxic-induced responses.

Apart from dERR and dHIFa, our data indicate that at least one more hypoxic-sensitive pathway is active and important for mediating hypoxic adaptation, as we found many H-sensitive transcripts that fall outside the regulation of either factor. The nature of the alternate pathway(s) is unknown. The results shown here suggest that identifying the sensors and effectors that regulate these HIF- & ERR-independent hypoxic response pathways will have profound impacts on our understanding of hypoxic signaling, and will undoubtedly provide new avenues with which to approach the complex problem of metabolic transition.

## Materials and Methods

### Fly strains and hypoxic treatments

Flies were maintained on regular cornmeal-molasses-yeast media at 25°C. *sima* mutants (*sima^07607^*) [43] were obtained from Bloomington Stock Center. *w^1118^* animals were treated as controls. *dERR* mutants (*dERR^1^/dERR^2^*) are described elsewhere [32]. *dERR,sima* double-mutants were generated by recombination of the *sima^07607^* allele with each of the individual *dERR^1^* and *dERR^2^* mutations. Embryos were collected at 25°C for 14 hrs onto egg caps (molasses-agar media in 35 mm×10 mm dishes) with yeast paste. Mid-L2 larvae were transferred to a fresh egg cap with blue yeast paste (0.3% bromophenol blue), and allowed to develop until achieving the partial clear-gut L3 stage (−10 to −4 hrs RTP). Staged animals were moved to fresh agar plates and allowed to age an additional 6 hours at 25°C (normoxic treatment); or, animals were placed in an airtight Modular Incubator Chamber (Billups-Rothenberg, Inc., Del Mar, CA) for 6 hours at 25°C after a gas mixture containing 4% oxygen balanced with nitrogen was flashed into the chamber (hypoxic treatment). The *sima^07607^* chromosome was carried over a TM3, twi-GFP (green fluorescent protein) balancer chromosome. Homozygous mutant larvae were sorted for the absence of GFP expression using a Zeiss Discovery V.8 dissecting stereoscope with fluorescence at mid-L2. For lethal phase analysis in Figure 6B, 0–4 hr post-hatch L1 larvae were sorted for fluorescence to assign genotype. Larvae were placed in vials containing fresh yeast paste and were then exposed to 21% (normal air) or 4% oxygen for 48 hrs and scored for lethality or completion of L1.

### Microarray analysis

Microarray analyses were performed on at least three biological replicates of *w^1118^* animals, *sima* mutants, *dERR* mutants, and *sima,dERR* double-mutants at the partial clear-gut L3 stage and treated for 6 hrs in normoxia or 4% O_2_. For each biological replicate, at least 10 larvae were collected and washed with 1×PBS before homogenization in TRIzol (Invitrogen, Carlsbad, CA) using a VWR disposable pellet mixer. Total RNA was isolated using a TRIzol/RQ1 DNase hybrid extraction protocol (Promega, Madison, WI). Template labeling was done using the GeneChip 3′ IVT Express Kit according to the manufacturer's specifications (Affymetrix, Santa Clara, CA). Hybridizations to Affymetrix GeneChip Drosophila Genome 2.0 arrays were performed using the manufacturers recommendations. Every chip was scanned at a high resolution by the Affymetrix GeneChip Scanner 3000 according to the GeneChip Expression Analysis Technical Manual procedures (Affymetrix, Santa Clara, CA). Raw data were normalized with RMA [62] and analyzed with the significance analysis of microarray (SAM) program [63]. No changes below 1.5-fold were considered significant. Additionally, the following false discovery rate percentages were imposed: 0.733% for *w^1118^* normoxia vs. *w^1118^* hypoxia; 0.414% for *sima* normoxia vs. *sima* hypoxia; 0.721% for *w^1118^* hypoxia vs. *sima* hypoxia; 7.84% *dERR* normoxia vs. *dERR* hypoxia; 0.619% for *w^1118^* hypoxia vs. *dERR* hypoxia; 0.662% *dERR,sima double-mutant* normoxia vs. *dERR,sima double-mutant* hypoxia; 0.703% for *w^1118^* hypoxia vs. *dERR,sima double-mutant* hypoxia. Microsoft Access was used to compare data sets. Microarray data from this study can be accessed at the Omnibus website (http://www.ncbi.nlm.nih.gov/geo) with the accession number GSE33100.

### Quantitative RT–PCR

Total RNA samples were isolated as described above. RNA was reverse transcribed with the High Capacity cDNA Reverse Transcription Kit (Applied Biosystems, Carlsbad, CA) using the manufacturer's specifications. For real-time PCR, premixed primer-probe sets were purchased from Applied Biosystems, with the exception of the primer set used for *amylase*. For *amylase*, a standard SYBR Green (Bioline, Taunton, MA) protocol was used with the primer sets: 5′ AACTACAACGACGCCAACGAG 3′ and 5′ TGGTCGGTGTTCAGGTTCTTG 3′. All amplifications were carried out on a CFX96 real-time PCR system (Bio-Rad, Hercules, CA). Experimental values were normalized to values obtained for the *Rp49* probe set. Data are reported as the mean±SEM. All values reported represent experiments performed on at least three biological replicates.

### Metabolic analyses

Analyses were performed on partial clear-gut L3 larvae treated for 6 hours in normoxia or 4% O_2_. After treatment, animals were washed twice in PBS pH 8.0 and immediately frozen at −80°C. For glycogen measurements, 45 animals were split into three pools and the assay was performed essentially as described [64]. Color intensity was measured using a Bio-Tek Elx800 absorbance micro-plate reader at 540 nm. Glucose and glucose+glycogen amounts were determined using a standard curve. The amount of glycogen was determined by subtracting the glucose from the glucose+glycogen total. Glycogen amounts were normalized to protein content in each homogenate using a Bradford assay (Bio-Rad). For ATP measurements, larvae were homogenized in 300 µl of 6M guanidine-HCl extraction buffer (100 mM Tris and 4 mM EDTA, pH 7.5). The homogenate was heated at 70°C for 5 min and centrifuged in at 3000×g for 1 min. The supernatant was diluted 1∶750 in dilution buffer (25 mM Tris and 100 µM EDTA, pH 7.5) and spun at 14000×g for 3 min, after which 10 ll supernatant was transferred to a 96-well white opaque plate and mixed with 100 ll of luminescent solution (Invitrogen, Molecular probes). Luminescence was immediately measured by a Bio-Tek Synergy 2 SL luminometer. The amount of ATP was determined using a standard curve. Amounts were normalized to total protein. For lactate measurements, 300 first instar larva, 60 third instar larva or 30 1-day-old males were split into three pools and measured as described Monserrate et al. (2012) using Lactate Assay Kit (Biovision Milpitas, CA,) [65]. For metabolomics, analyses were performed by Metabolon, Inc. (Durham, NC). Replicates were normalized by protein content (Bradford analysis). Recovery standards were added to samples prior to extraction using a proprietary series of organic and aqueous solutions. Extracts were divided into two fractions, one for GC and one for LC. Organic solvent was removed using a TurboVap (Zymark). Briefly, for LC/MS, split samples were dried and reconstituted in acidic or basic LC-compatible solvents containing standards. Positive and negative ion-optimized sample conditions were analyzed in separate injections. For acidic reconstitutions a gradient of water and methanol containing 0.1% formic acid was used, and for basic extracts a water/methanol gradient with 6.5 mM NH_4_HCO_3_. Analysis was performed on a Thermo-Finnigan LTQ mass spectrometer with an electrospray ionization source and linear ion-trap mass analyzer. For GC, samples were re-dried under vacuum prior to derivatization under nitrogen using bistrimethyl-silyl-trifluoroacetamide. The column was 5% phenyl with a temperature ramp of 40° to 300°C over 16 minutes. Samples were analyzed using a Thermo-Finnigan Trace DSQ fast-scanning single-quadrapole mass spectrometer with electron impact ionization. Refer to Table S2 for normalized data of each replicate and *p*- and *q*-values. Extensive quality control care was applied to minimize variability between days. The Metabolon platform has been described elsewhere [66], [67]. Data values were imputed in the following way when values fell below the threshold level of detection: when all six replicates were undetectable, each was assigned the minimum detectable value of across all compounds tested; when five or less replicates were undetectable, sample values were assigned the minimum value obtained among those that were detected for a given compound.

### Yeast two-hybrid screen and GST-pulldown

A yeast two-hybrid screen was conducted using the Invitrogen ProQuest Two-Hybrid System. For this purpose, three cDNA prey libraries were simultaneously prepared using the CloneMiner cDNA Library Construction Kit (Invitrogen). All the libraries (a, b, c) were made from poly-A-selected RNA that was extracted from *w^1118^* animals at −4, +0, or +4 RTP, which was reverse transcribed and pooled in equal proportions before library construction. Each library differs by only a single base pair in the adapter sequence to facilitate expression of clones in all three frames. Extensive procedures, provided by the manufacturer, were followed to capture clones into the pDONR222 vector. Clones in the donor vector were subsequently recombined into the pDEST22 vector. Libraries were titered (a = 7.18E6 CFU, b = 4.44E6 CFU, c = 14.28E6 CFU) and sampled for average insert size (a = 1.64 kb, b = 1.25 kb, c = 1.6 kb) before transformation into ElectoMax cells (Invitrogen). Transformed cells for each library were pooled (total of 6.4E6 CFU) and grown for 22 hrs at 30°C for preparation of library DNA by standard techniques. 22lg of library DNA was transformed into the yeast bait strain containing the LBD of dERR (L193-R496) that had been recombined into the pDEST32 vector. A total of 5.28E5 clones were screened by auxotrophic selection. All positive hits were sequenced. GST-pulldown experiments and the expression of GST-fused ERR constructs in pGEX-4T1 were performed as described [68].

### Statistical analysis

A one-way ANOVA *F*-test was applied to test for the differences in glycogen levels, followed by Tukey's HSD method. For developmental qRT-PCR analysis, delta CT values were used to perform statistical analysis, whereby a two-tailed unpaired student's t-test was applied for the differences in gene expression using a Bonferroni correction. Following log transformation and imputation, a one-way ANVOA with contrasts was used to identify significance for metabolites in the mass spec analysis (See Table S2). Cumulative hypergeometric probability was used to determine significance between overlapping gene sets.

## Supporting Information

Figure S1 Scheme to identity HIF-independent (HI) and HIF-dependent (HD) hypoxia-regulated genes. The HIF-independent set (640 genes), represents the direct comparison of N- or H-treated samples from *sima* mutants. The Total H-genes set (1127 genes) represents the direct comparison of N- or H-treated samples from *w^1118^* animals. The HI H-genes set (black circle), represents the overlap of the Total H-genes set with the dHIF-independent set. The dHIF-dependent set2 (873 genes), represents the subtraction of the dHIF-independent H-genes set from the Total H-genes set. The dHIF-dependent set1 (801 genes) represents the direct comparison of H-treated samples from *w^1118^* animals and *sima* mutants. The HD H-genes set (green circle), represents the overlap between the dHIF-dependent sets1 and 2. All genes in any of the sets are up- or downregulated at least 1.5-fold and have a FDR of less than 1%. See Table S1 for gene set lists.
(EPS)

Figure S2 Scheme to identity ERR-dependent (ED) and ERR&HIF-dependent (DM) hypoxia-regulated genes. The HD H-genes (green circle) were determined as outlined in Figure S1. The dERR-independent set (blue outlined oval) represents the direct comparison of N- or H-treated samples from *dERR* mutants. The Total H-genes set (black outlined oval) represents the direct comparison of N- or H-treated samples from *w^1118^* animals. The dERR-dependent set2, represents the subtraction of the dERR-independent set from the Total H-genes set. The dERR-dependent set1 represents the direct comparison of H-treated samples from *w^1118^* animals and *dERR* mutants. The ED H-genes set (blue circle), represents the overlap between the dERR-dependent sets1 and 2. The ERR&HIF-dependent H-genes set (red circle) was determined using the same scheme as above, except that the *dERR,sima* double-mutant was used instead of the *dERR* mutant. All genes in any of the sets are up- or downregulated at least 1.5-fold and have a FDR of less than 1%, except for the dERR-independent set (noted with *). FDR constraints were relaxed for this set alone. It includes FDR scores that are elevated to <7%. See Table S1 for gene set lists.
(EPS)

Figure S3 Normoxic expression versus hypoxic induction of HIF-dependent transcripts. qRT-PCR analysis of transcripts shown in Figure 3. Traces are the temporal expression profiles for each transcript in normoxia or hypoxia (treated for 6-hr in 4% O_2_) from *w^1118^* animals or *sima* mutants. Values were arbitrarily scaled to the value obtained for 6–12 hr embryonic time-point for the *w^1118^* control in normoxia, which was assigned a value of 1.0. All values are normalized to *rp49* expression. Error bars are the SEM.
(EPS)

Figure S4 Normoxic expression versus hypoxic induction of HIF-independent transcripts. qRT-PCR analysis of transcripts shown in Figure 4. Traces are the temporal expression profiles for each transcript in normoxia or hypoxia (treated for 6-hr in 4% O_2_) from *w^1118^* animals or *sima* mutants. Values were arbitrarily scaled to the value obtained for 6–12 hr embryonic time-point for the *w^1118^* control in normoxia, which was assigned a value of 1.0. All values are normalized to *rp49* expression. Error bars are the SEM.
(EPS)

Figure S5 Lactate production in hypoxia is life-stage-dependent. Lactate measurements from mid-L1 (A), late-L3 (B), and day-old males (C) from *w^1118^* animals and *sima* mutants treated for 0, 4, or 6 hrs in 4% O_2_. All measurements were determined in triplicate. Values are normalized to protein content. Error bars are the SEM.
(EPS)

Figure S6 Gene ontology analysis of ERR-dependent and ERR&HIF-dependent hypoxic genes. GO analysis was performed on the dERR-dependent (ED) genes set (A) and on the dERR&dHIF (DM) genes set (B) that that were derived from microarray analysis of H- or N-treated control (*w^1118^*) animals, *ERR* mutants or *dERR,sima* double-mutants collected at the partial clear-gut late-L3 time. See Figure S2 for the analysis scheme. The numbers of H-regulated genes affected are shown along with the total number of genes in each category. All transcripts are up- or downregulated at least 1.5-fold and have a false discovery rate (FDR) of <1%.
(EPS)

Table S1 Hypoxia-regulated gene sets identified by microarray analysis.
(XLS)

Table S2 Metabolic analysis of carbohydrates by GC/MS and LC/MS.
(XLS)

Table S3 Lethal phase analysis of embryos and L1 larvae subject to different oxygen concentrations.
(XLS)
